# Multi-method assessment of whale shark (*Rhincodon typus*) residency, distribution, and dispersal behavior at an aggregation site in the Red Sea

**DOI:** 10.1371/journal.pone.0222285

**Published:** 2019-09-09

**Authors:** Jesse E. M. Cochran, Camrin D. Braun, E. Fernando Cagua, Michael F. Campbell, Royale S. Hardenstine, Alexander Kattan, Mark A. Priest, Tane H. Sinclair-Taylor, Gregory B. Skomal, Sahar Sultan, Lu Sun, Simon R. Thorrold, Michael L. Berumen

**Affiliations:** 1 Red Sea Research Center, Division of Biological and Environmental Science and Engineering, King Abdullah University of Science and Technology, Thuwal, Kingdom of Saudi Arabia; 2 Massachusetts Institute of Technology–Woods Hole Oceanographic Institution Joint Program in Oceanography/Applied Ocean Science and Engineering, Cambridge, MA, United States of America; 3 Biology Department, Woods Hole Oceanographic Institution, Woods Hole, Massachusetts, United States of America; 4 Centre for Integrative Ecology, School of Biological Sciences, University of Canterbury, Private Bag, Christchurch, New Zealand; 5 Marine Spatial Ecology Lab, School of Biological Sciences, University of Queensland, St. Lucia, Queensland, Australia; 6 Massachusetts Division of Marine Fisheries, New Bedford, MA, United States of America; 7 School of Biology, University of St Andrews, St Andrews, Scotland, United Kingdom; 8 Key Laboratory of Science and Engineering for Marine Ecology and Environment, First Institute of Oceanography, Ministry of Natural Resources, Qingdao, China; Institut de Recherche pour le Developpement, FRANCE

## Abstract

Whale sharks (*Rhincodon typus)* are typically dispersed throughout their circumtropical range, but the species is also known to aggregate in specific coastal areas. Accurate site descriptions associated with these aggregations are essential for the conservation of *R*. *typus*, an Endangered species. Although aggregations have become valuable hubs for research, most site descriptions rely heavily on sightings data. In the present study, visual census, passive acoustic monitoring, and long range satellite telemetry were combined to track the movements of *R*. *typus* from Shib Habil, a reef-associated aggregation site in the Red Sea. An array of 63 receiver stations was used to record the presence of 84 acoustically tagged sharks (35 females, 37 males, 12 undetermined) from April 2010 to May 2016. Over the same period, identification photos were taken for 76 of these tagged individuals and 38 were fitted with satellite transmitters. In total of 37,461 acoustic detections, 210 visual encounters, and 33 satellite tracks were analyzed to describe the sharks’ movement ecology. The results demonstrate that the aggregation is seasonal, mostly concentrated on the exposed side of Shib Habil, and seems to attract sharks of both sexes in roughly equal numbers. The combined methodologies also tracked 15 interannual homing-migrations, demonstrating that many sharks leave the area before returning in later years. When compared to acoustic studies from other aggregations, these results demonstrate that *R*. *typus* exhibits diverse, site-specific ecologies across its range. Sightings-independent data from acoustic telemetry and other sources are an effective means of validating more common visual surveys.

## Introduction

The whale shark *Rhincodon typus* (Smith 1828) is a large-bodied, epipelagic, filter feeder [1]. The species is cosmopolitan in tropical and warm temperate waters, though its diffuse distribution has historically hindered both scientific study and conservation efforts. While *R*. *typus* is still frequently described as enigmatic, the discovery of high density, predictable aggregations has sparked a rapid expansion in research on this species [2–15]. In addition to their value as study sites, these aggregation areas have often become an ecotourism attraction and an economic boon to local communities [16–18]. Understanding the population dynamics, seasonality, and movement ecology of each site is vital for researching and sustainably managing these valuable natural resources.

Since their discovery, aggregations of *R*. *typus* have typically been described using visual census and photo-identification [6, 9, 12, 19–24]. Cooperation among research groups, tour operators, and citizen scientists has produced an extensive record of *R*. *typus* encounters, much of which has been collected in a single online database (www.whaleshark.org). A 22-year overview of this aggregate dataset encompassed nearly 30,000 documented encounters with 6000 individual *R*. *typus* from 54 countries [25]. This global record has helped define the typical aggregation as a collection of mostly juvenile males which gather seasonally to exploit ephemeral food sources. Smaller, more localized studies have used visual census to track patterns of habitat use within aggregations [26, 27], to measure connectivity between them [24], and to describe exceptional sites which either attract unusual demographics [12, 28] or have aseasonal patterns of *R*. *typus* presence [7, 21].

Collaboration and the amount of available data have made visual census a powerful tool, but it has limitations. First, dedicated search efforts are largely confined to known aggregations. Outside of these areas, researchers have had to rely on encounter records from pelagic fishermen [29] or satellite tracking data from relatively small samples of tagged sharks [3, 13, 15, 23, 30–38]. Second, even within aggregations, boat-based surveys are often restricted to the surface and the ability to reliably find sharks declines significantly at night, in rough seas, or when the targeted animals are at depth. Search effort may also be restricted in areas where research or ecotourism are confined to specific “field-seasons.” Because of these limitations, the absence of encounter data may be a poor proxy for absence of *R*. *typus*. To account for this, researchers have begun to incorporate sightings-independent data into their site descriptions, and these data have not always agreed with the results of visual surveys [14, 39].

For instance, at Mafia Island, Tanzania and Ningaloo Reef, Australia sightings records have been compared to data from concurrent passive acoustic monitoring, a method which uses fixed listening stations to record the presence of animals tagged with acoustic transmitters [14, 39]. In both cases, visual census methods showed strong seasonal patterns that were not observed in the passive acoustic data. The authors concluded that seasonal lulls in sightings frequency corresponded either to small-scale shifts in the sharks’ habitat selection [14] or to reductions in search effort [39] rather than migration. Acoustic studies on *R*. *typus* are still uncommon, so the combination of visual surveys with comparable sightings-independent data is not yet available for most aggregations. Because of this, it is unclear whether the cryptic residency shown at Mafia Island and Ningaloo Reef is prevalent elsewhere. In addition, both Mafia and Ningaloo host male-dominated aggregations [40, 41], so passive acoustic monitoring of females is particularly lacking.

Visual census [28] and satellite telemetry [13] data have revealed a juvenile *R*. *typus* aggregation at Shib Habil—a coastal reef in the Saudi Arabian Red Sea. The available data suggests that this aggregation has well-defined seasonal structure and unusual sexual demographics in which *R*. *typus* of both sexes aggregate during the boreal spring months of March, April, and May [13, 28]. In the present study, six years of passive acoustic monitoring at this site are analyzed and compared to published visual [28] and satellite [13] data collected from the same individual sharks, over the same period. Collectively, these data are used to describe the residency behavior, seasonal philopatry, and spatial distribution of aggregating sharks, as well as to investigate the apparent sexual integration found at this site.

## Methods

### Ethics statement

The King Abdullah University of Science and Technology (KAUST) operates all marine research under a broad permit from the Kingdom of Saudi Arabia. Additionally, all vessels (including research vessels) must obtain permission to leave port from the Saudi Arabian Coast Guard. Similarly, all vessels must report back to the Coast Guard and submit to a search before returning to port. In order for this research to be carried out under KAUST's general permit, all procedures needed to be approved by the Institutional Biosafety and Bioethics Committee (IBEC). KAUST IBEC serves as the registered (HAP-02-J-042) local committee for all National Committee of Bioethics (NCBE)-regulated activities including animal-related research.

While the whale shark was declared endangered in 2016 and all sharks are protected from fishing within Saudi Arabian Waters, the research presented here does not violate those protections. No animals were sacrificed, collected, or restrained over the course of this study. All procedures were conducted on free-swimming sharks in their natural environment. Under these circumstances, it was determined that no additional permissions were required beyond KAUST's general permit, IBEC approval, and authorization to leave port.

### Data collection

**Fig 1:**
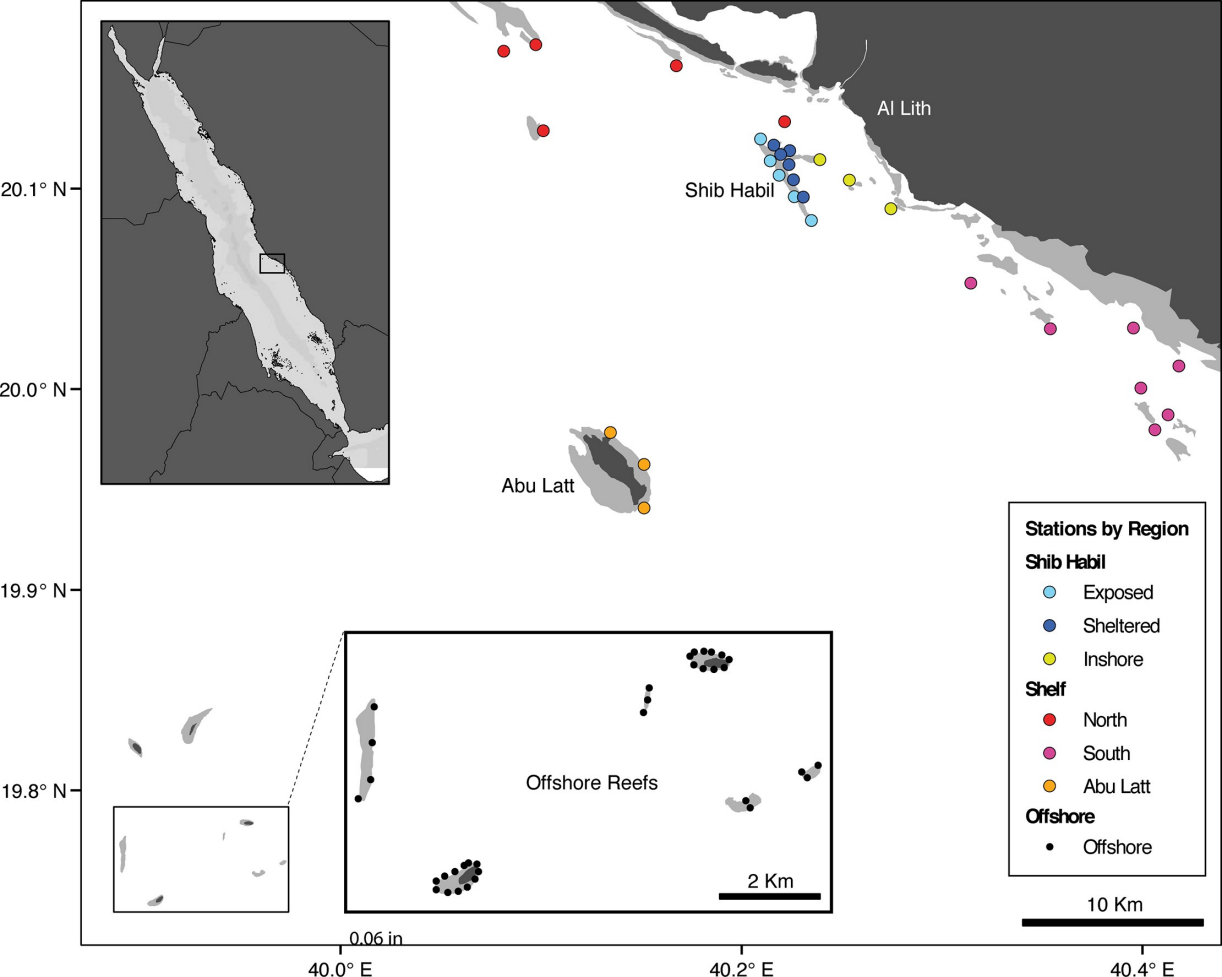
Map of the acoustic array. Top-left inset shows the position of Shib Habil within the Red Sea. Bottom-center inset provides a zoomed in view of the offshore array. Receiver stations are represented by point markers and are colored to show the regional divisions within the array as indicated by the legend in the bottom right.

Beginning in March 2010, 63 stationary acoustic receivers (Model VR2W, Vemco LTD., Halifax, Canada) were deployed in the Al Lith area (Fig 1). These stations were grouped into seven geographic regions: the exposed side of Shib Habil (5 stations), the sheltered side of Shib Habil (6 stations), inshore of Shib Habil (3 stations), the northern continental shelf (4 stations), the southern shelf (7 stations), the outer-shelf island of Abu Latt (3 stations), and the offshore reefs (34 stations). Independent range tests were performed at Shib Habil (nominal 50% detection range of 540 m) and at offshore receivers (230 m) [42]. The array was downloaded and stations maintained between two and three times per year on average.

Externally-cased, individually-coded acoustic transmitters (V16 and V16P 6H, 69 kHz, random delay 60–180 s, Vemco LTD., Halifax, Canada) were tethered to an intramuscular titanium anchor (Wildlife Computers, Inc., Seattle, USA) using stainless steel wire (covered in heat-shrink wrap to keep the wire from abrading the shark’s skin). Free swimming *R*. *typus* were approached by snorkelers who used sling-spears to insert the intramuscular anchors into the base of the shark’s first dorsal fin. During tagging, snorkelers visually estimated total length and determined sex by observing the presence or absence of claspers between each shark’s pelvic fins. Size and (in males) clasper morphology were used to estimate the broad life-stage (juvenile/mature) of each animal [12,40,41]. From March 2010 through April 2016, 106 acoustic tags were deployed on 97 individuals (39 females, 43 males, and 15 sharks of undetermined sex). Nine sharks (six females, three males) shed their initial transmitters and were retagged on subsequent trips. One tag was recovered from a dead specimen (bycaught in a gill net by a local fisherman) and later redeployed. Transmitter deployments were not evenly distributed among years and depended on the frequency of untagged shark encounters as well as the number of available tags. In total, 37 transmitters were deployed in 2010, 39 were deployed in 2011, 15 in 2012, 5 in 2014, 10 in 2015, and 11 in 2016. All tagging was opportunistic (i.e. we did not reserve tags to target particular demographics) and occurred during the purported high-season between the beginning of March and the end of May of each year.

Publically available photo-identification records [28] (www.whaleshark.org) and satellite tracking data [13] from Shib Habil were accessed and searched for acoustically tagged sharks from the present study. In total, 28 sharks were fitted with all three tag types (photographic, acoustic, and satellite), 48 had acoustic and photographic tags only, and 10 had acoustic and satellite tags only. Of the 38 sharks with satellite tags, eight were fitted with Non-archival Argos transmitters (Model SPOT5, Wildlife Computers, Inc., WA, USA) while the remaining thirty were fitted with Pop-up Satellite Archival Transmitting (PSAT) tags (Models Mk10-PAT and Mk10-AF; Wildlife Computers, Inc., WA, USA). While at the surface, both tag types are capable of acquiring Doppler-based position estimates through communication with Argos satellites. In addition, the PSAT tags also log temperature, depth, and light-level data which can be used to calculate daily geolocation estimates even when the tagged animals are submerged. Additional information for both the photo-identification and satellite telemetry (including detailed field methods) can be found in the original publications [13, 28].

### Data analysis

Acoustic records were filtered by tag number and deployment times so as to only include detections of tagged *R*. *typus* (S1 Appendix). In addition, Vemco VR2W receivers are prone to internal clock drift, so known initialization and download times were used to correct for possible temporal discrepancies. Over the course of the study, several receiver units were lost and either replaced or the site was abandoned. The resulting fluctuations in monitoring effort were tracked and accounted for during data analysis either by including receiver effort as a modeled variable or by treating a given station’s unmonitored days as undefined (the days monitored for each of the 63 stations can be found in S1 Table). Similarly, several sharks were eventually resighted after having lost their transmitters and one shark is known to have died. In these cases, the sharks were assumed to have lost their tags immediately after the last recorded detection. Finally, transmitter attachment by subdermal injection may be stressful for the animal and could temporarily alter its behavior. To avoid analyzing potentially unnatural movement patterns, all acoustic detections of an individual collected within 24 hours of tag application were not included in the analysis. In total, 13 individuals were removed from the analyzed dataset, leaving 84 sharks (35 females, 37 males, 12 undetermined).

Many passive acoustic studies, including those targeting *R*. *typus*, have used detection data to produce some form of residence index [14, 39, 43–49]. This is usually calculated as the number of days an animal was detected divided by the number of days it was monitored, though the exact definition of days-monitored has varied. For instance, Cagua et al. [14] used a conservative index that calculates days-monitored as the period between tagging and the end of the study. This definition assumes that once deployed, tags will remain functional and attached indefinitely, creating a maximum monitoring period and a minimum residence index (R_min_). Conversely, Norman et al. [39] used a residence index that accounted for tag-losses by defining days-monitored as the period between tagging and final detection. This definition creates a minimum monitoring period and a maximum residence index (R_max_). Neither of these indices (R_min_ or R_max_) is strictly correct because both are directly affected by study duration, which can bias values upward for animals that were tagged later (in the case of R_min_) or detected over shorter periods of time (in the case of R_max_). In the present study, both indices were calculated in order to facilitate comparison with earlier research, to compare results between the two metrics, and to provide upper and lower bounds for each animal’s true residency behavior.

In addition to calculating the residence indices, we also fit a series of generalized additive mixed-effects models (GAMMs) to both the visual and acoustic detection histories [14] (S1 Appendix). Both datasets were divided into six-week bins, and each shark’s presence/absence was modeled as a per-individual, binomial occupancy-metric defined as one if the shark was resighted/detected during a given time-bin and zero if the shark was not. The occupancy metric was then logit-linked to a series of explanatory variables. These included two smooth terms: temporal lag (quantified as the number of days between all potential capture events for each shark and included in the model as a low rank isotropic smoother) and time of year (quantified as week of the year and included in the model as a cyclic cubic regression spline). The model also used several fixed terms, including the size and sex of tagged *R*. *typus*, survey effort (for visual census), and the number of inshore/offshore receivers active within the array at any given time (for acoustic monitoring). Finally the model included two random effects in addition to a binomial error structure: shark identification numbers were used to account for non-independence of data from the same individual shark and the date of initial capture was used to account for pseudo-correlation caused by calculating every possible value for temporal lag. Models were fitted for all combinations of explanatory variables that included the smooth terms (temporal lag and time of year), totaling sixteen candidate models for the acoustic detection record (acoustic GAMMs) and eight for the visual census data (visual GAMMs) (all candidate models are listed in S2 Table). Models were fitted using the mgcv 1.8–27 package of the R programming language [50] and selected using the Akaike Information Criterion (AIC). The selected acoustic and visual GAMMs to estimate the significance of each modeled parameter (S3 Table) and to estimate the odds of recapture. Finally, the recapture odds for all parameters were summed together with the intercept and subjected to an inverse-logit function to derive acoustic and visual recapture probability curves accounting for all modeled variables.

Spatiotemporal patterns in *R*. *typus* distribution were quantified by comparing detections per unit effort among receiver stations and by constructing a spatially explicit variant of the residence index. Spatial residence (R_spatial_) was calculated as the number of days a specific tagged shark was detected at a given station divided by the number of days it was detected within the array as a whole. To compensate for gaps in monitoring effort, days in which a station was inactive due to receiver malfunction or loss were excluded from the index calculations for that station. For instance, if a shark was detected on two days at a given station and on four days within the array as a whole, it would normally produce an index value of 2/4 (0.5) for that station. However, if the station was inactive on one of the days where the shark was detected in the array, that day would be excluded from the calculation resulting in an index value of 2/3 (0.67). Average R_spatial_ values were calculated for each station using the results from all tagged sharks. Male and female index values were also averaged separately for each station and compared using Mann Whitney U tests. The number of comparisons (63, one for each station in the array) increases the likelihood of a Type I error (an apparently significant result occurring by chance without reflecting an actual difference between the sexes). In order to account for this problem of multiple comparisons, apparently significant results (based on α = 0.05) were also checked against a Bonferroni corrected critical value of 0.0008 (calculated as the standard α divided by the number of comparisons or 0.05/63).

Finally, satellite tag data [13] was re-analyzed to incorporate the additional “known” location information derived from acoustic monitoring and visual census. For sharks tagged with SPOT tags, Argos positions were assigned error classes (Z, B, A, 0, 1, 2, 3) to reflect position accuracy. Positions assigned error class Z (unknown accuracy) and locations on land were discarded. Remaining Argos locations were speed filtered using a 4 m/s maximum speed cutoff to remove extraneous positions. Daily acoustic and visual detections were added to the Argos data as class 2 (accurate to 500m) and 3 (250m) positions, respectively. Estimated tracks were constructed for PSAT-tagged sharks using a hidden Markov model (HMM) built by the tag manufacturer (WC-GPE3, Wildlife Computers) following the methods of Skomal et al. [51] and incorporating the acoustic and visual detection data as “known” daily positions. This approach uses a gridded HMM that computes posterior probability distributions to estimate the most likely state (position) at each time point based on in-situ light levels, sea surface temperature and depth constraints recorded onboard the tag. Recent work has suggested position estimates using this approach for surface-oriented species with moderate to high quality datasets is ~80–150 km (root-mean-square track error) [52].http://www.ngdc.noaa.gov/mgg/global/etopo2.html The most likely tracks based on the combined Argos, archival, acoustic, and visual data were then mapped for each animal and used to characterize individual migration behaviors.

## Results

### Residency and seasonal structure

The analyzed dataset consisted of 37,461 detections of 84 sharks. The tagged population ranged in size from 3–7 meters total length with an overall average of 4 meters (females 4.1, males 3.9) (S4 Table). Based on these size estimates and the immature clasper morphology observed in males, all tagged shark were classified as juveniles. Acoustic records showed high individual variation in detection counts (range: 4–3995), total days recorded within the array (range: 1–265), and minimum monitoring periods (range: 2–2216). Seventeen sharks were tracked for fewer than 10 days, recording an average of 2.9 days within the array (range: 1–7) and 61.4 detections per individual shark (range: 9–166). At the other extreme, 28 sharks were tracked for more than a year, averaging 43.1 days within the array (range: 2–265) and 833.9 detections (range: 11–3995). High individual variation was also apparent in both residence indices, even when comparing animals from the same tagging cohort (for R_min_) or with similar tracking histories (for R_max_). The tagged population had an overall average R_min_ value of 0.05 (range 0–0.88). The 2010 tagging cohort tended to have lower R_min_ values (mean: 0.01, range: 0.00–0.11), while those from 2016 trended higher (mean: 0.55, range: 0.23–0.88). Maximum residence (R_max_) ranged from 0.00 to 1.00 with an average of 0.26. Sharks detected in only one calendar year had higher R_max_ values (mean: 0.48, range: 0.03–1.00) than those monitored over multiple years (mean: 0.05 range: 0.00–0.22).

Female sharks recorded more detections (603.1 per individual, range: 4–3781) and a greater number of days within the array (26.0 per individual, range: 2–265) than did males (398.6 detections and 18.2 days per individual, ranges: 5–3995 and 1–115 respectively), though these differences were not statistically significant at α = 0.05 (detections per individual; Mann Whitney Test, U = 564.5, p = 0.23) (days per individual; Mann Whitney Test, U = 564.0, p = 0.23). Residence indices were also similar between the sexes (Mann Whitney Test, U = 607.5–697.5, p = 0.400–0.483). Males averaged 0.06 for R_min_ and 0.24 for R_max_ while females averaged 0.07 and 0.28 respectively. In addition to the similar residency patterns, the array also revealed a high degree of overlap between male and female presence. Over the six-year study period, sharks of known sex were recorded within the array on 657 days, including 336 days with multiple such individuals. There were 151 days with only male detections (including 23 days with multiple males), 242 days with only female detections (49 with multiple females), and 264 days in which tagged sharks of both sexes were detected.

**Fig 2:**
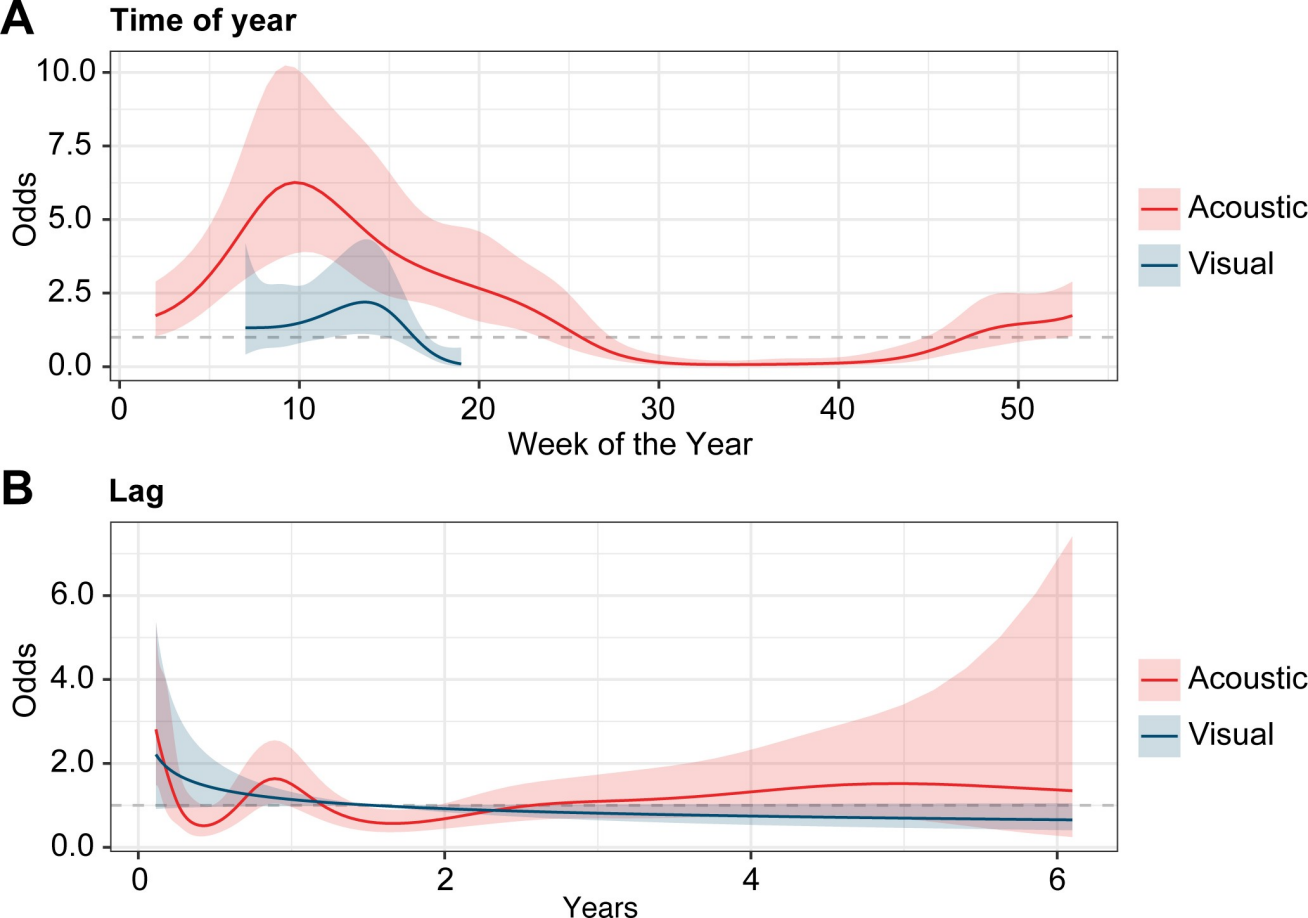
Visual and acoustic recapture odds vs time of year and temporal lag. The line graphs track the odds of recapture for the mixed models’ hypothetical “typical” and how those odds change with (A) time of year and (B) temporal lag. The dashed line represents the mean odds of recapture for both methods, putting the visual and acoustic data on the same relative scale. There are clear peaks for both methods in relation to time of year, though the visual census dataset is restricted to the spring months when surveys were conducted. Recapture odds are comparatively flat in response to temporal lag, indicating high interannual fidelity in at least a few sharks.

**Fig 3:**
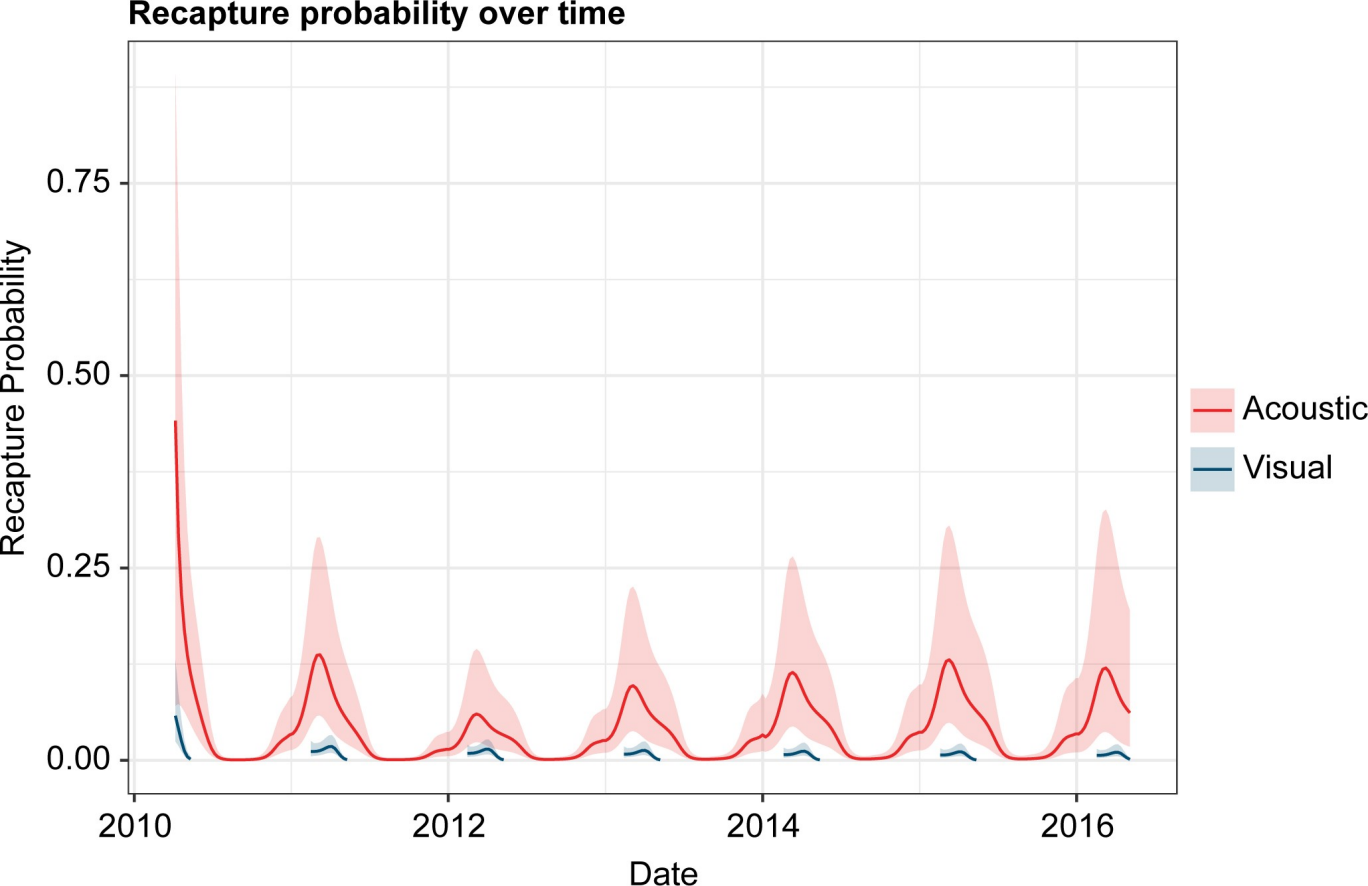
Visual and acoustic recapture probability over time. The line graph shows the mixed models’ daily estimate of visual and acoustic recapture probability for a hypothetical “typical” Shib Habil shark. The acoustic recapture profile is based on the assumption of maximum receiver effort throughout the study and both curves assume that the hypothetical shark was tagged/photographed in 2010 and is of average size (4 meters). Annual peaks in recapture probability are clear for both methods and occur at roughly the same time each year but are consistently higher in the acoustic model.

Despite the high individual variation in residency, the seasonal timing of *R*. *typus* presence was consistent throughout the study period and across the tagged population. The vast majority of detections (more than 98%) occurred in the first half of each year. Acoustic activity was relatively low in January (approximately 2% of total detections), increased in February (6%) and March (10%), and peaked in April (48%), or May (26%) before declining in June (3%). The sharks were mostly absent from July through December which, combined, accounted for less than 2% of total detections. This seasonal pattern of *R*. *typus* presence/absence was also apparent in the visual and acoustic mixed models. The 76 sharks with both acoustic detections and identification photos accumulated 35,243 acoustic detections along with 210 encounters in visual surveys. These data were used to fit several recapture models for both methods (S2 Table). The selected acoustic GAMM included time of year, lag, inshore receiver effort, offshore receiver effort, and animal size as parameters. With the exception of size (p = 0.25), all variables included in the selected model were found to have significant predictive value (p = 0.00–0.01). The selected visual GAMM produced similar results with time of year, lag, and animal size as parameters. As with the selected acoustic GAMM, time of year and lag were significant predictors of whale shark recapture (p = 0.01) while size was not (p = 0.51). The odds of both acoustic and visual recapture were most strongly affected by time of year (Fig 2A) with clear peaks in March and April respectively. The effect of lag was comparatively limited in both models (Fig 2B), though the odds of acoustic recaptures increased after approximately one year, indicating the annual periodicity of the aggregation. In both models, the combined effects of time of year and lag lead to annual cycles of high and low recapture probability that were fairly similar from year to year (Fig 3). Finally, neither model included sex as a parameter, suggesting that the sexes showed similar patterns of presence/absence within the array, regardless of the survey method.

### Spatial distribution

Acoustic records were not evenly distributed throughout the array (S1 Table). The twelve most active stations recorded a total of 35,571 detections (95% of the total dataset), or 1.83 per functioning receiver per day. The remaining 51 receivers only recorded 1890 detections, or approximately 0.05 per receiver-day; twelve of these stations recorded zero detections despite 8232 days of combined monitoring effort. The divide between the active and inactive portions of the array was also clear in the R_spatial_ index. Stations on Shib Habil’s exposed side had the highest index values (Mean R_spatial_: 0.145), followed by the northern shelf (0.052), and Shib Habil’s sheltered side (0.036). Stations inshore of Shib Habil, on the southern shelf, Abu Latt and on the offshore reefs all reported far lower index values (Mean R_spatial_: 0.003–0.013). The overall distribution of acoustic records largely reflects the distributions observed during the aggregation season, when the majority of detections were collected. Throughout the aggregation (January-June) and especially during the peak season, acoustic activity was highly concentrated along Shib Habil’s exposed side (5.09 detections per receiver-day), its sheltered side (1.88), and on the northern shelf (1.89). Over the same period, the sharks were mostly absent from stations inshore of Shib Habil (0.10 detections per receiver-day), on the southern shelf (0.30), near Abu Latt (0.11), or on the offshore reefs (0.02). During the offseason from July through December, detections were fewer but also more evenly distributed throughout the array. While stations inshore of Shib Habil were completely inactive during this period (0.00 detections per receiver-day) and activity on the northern shelf was comparatively high (0.09), the remainder of the array was fairly homogenous (0.016–0.026).

**Fig 4:**
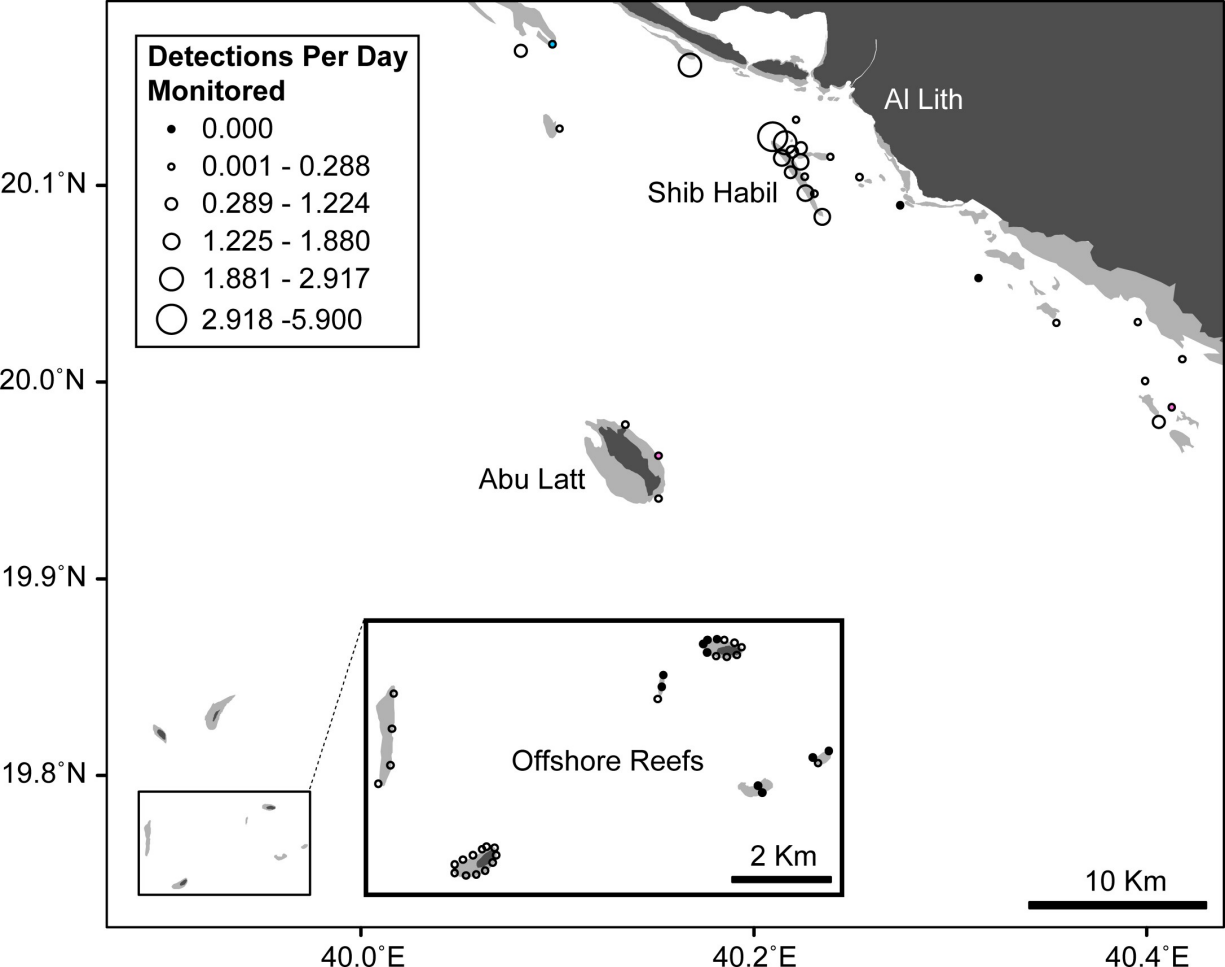
Map of the array showing detections per unit effort at each station. This map of the Shib Habil acoustic array uses graduated symbols to indicate detections-per-day monitored at each station (the twelve stations with zero detections are shown in black). Three stations reported significant sexual differences (at α = 0.05) in either detection counts or Rspatial values: one station (shown in blue) recorded higher values for males and two (shown in pink) recorded higher values for females. The remaining 60 stations reported similar values for both sexes.

Male and female sharks exhibited similar distributions throughout the array. Only two stations, one on the southern shelf (S7) and the other near Abu Latt (A2), recorded sexual differences in individual detection counts that were significant at α = 0.05 (Mann Whitney Test, U = 140–168, p = 0.031–0.039) with both reporting higher values for female sharks. The same station from the southern shelf (S7) and one on the northern shelf (N2) recorded significant (again at α = 0.05) sexual differences (Mann Whitney Test, U = 136–206, p = 0.02–0.021) in their R_spatial_ values, though the northern station reported higher values for males. Neither the female bias shown for S7 and A2 nor the male bias shown for N2 were significant at the Bonferroni corrected α = 0.0008, so it is plausible that all of these observations are actually Type I errors due to multiple comparisons. In addition, these three stations were not particularly active for either sex (Fig 4). Combined they recorded 298 detections (0.14 per receiver-day), accounting for only 0.8% of the total acoustic dataset. Given the low overall detection counts at these sites, it unlikely that any differences are ecologically significant compared to the similar male and female values (Mann Whitney Test, U = 28–613, p = 0.052–0.48) recorded at the remaining 60 stations, including all twelve of the array’s most active sites.

### Dispersal and philopatry

**Fig 5:**
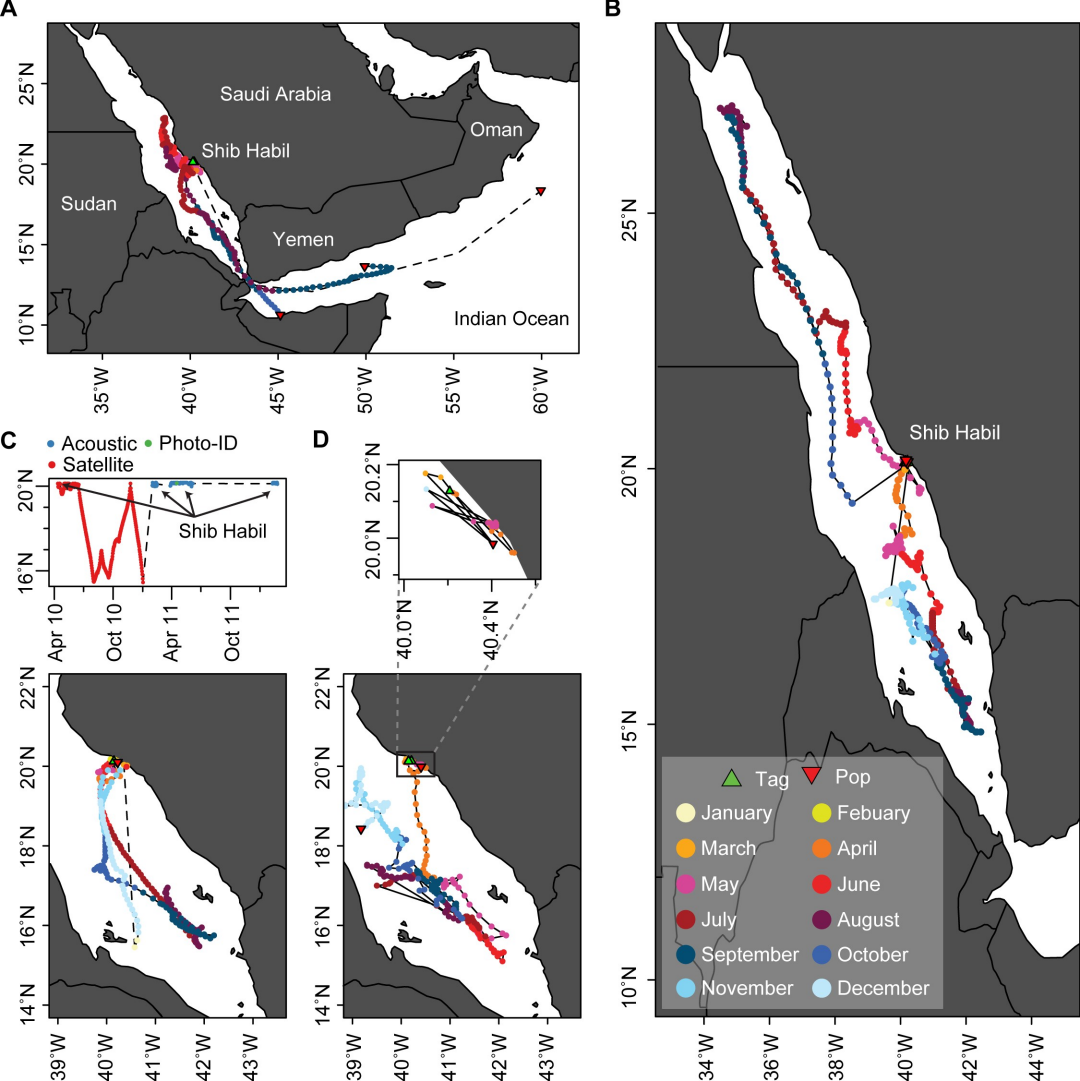
Reconstructed multi-method tracks for R. typus. Maps showing dispersal and migration behaviors of sharks tagged with both satellite and acoustic transmitters. Recorded behaviors included (A) Emigration from the Red Sea (three tracks shown out of three recorded in the study), (B) migrations away from and returning to Shib Habil (two tracks shown, 17 recorded), (C) Multiple return migrations (one track shown, four recorded), (D) apparent permanent emigration from Shib Habil (one track shown, 11 recorded), and (D, inset) no detected migration away from the study area (one track shown, three recorded).

**Fig 6:**
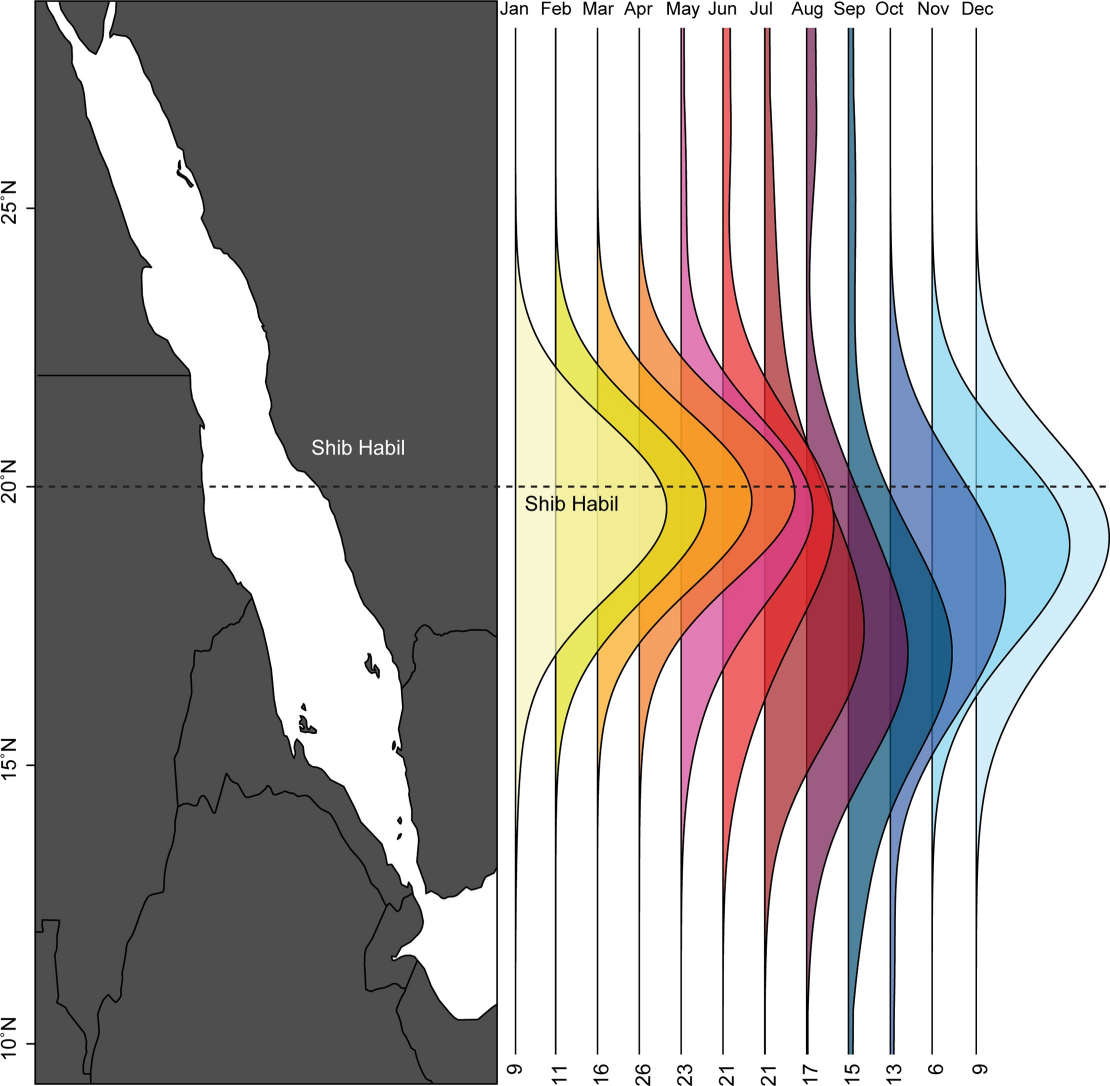
Latitudinal distribution of multimethod tracking data. This stacked data density plot shows the latitudinal distribution of multimethod tracking data. Each vertical line represents a month of the year and the numbers below each line indicate the number of sharks tracked in that month. Data is highly concentrated around Shib Habil during the aggregation season (January-June) but disperses (mostly southward) during the off-season.

Of the 76 sharks with both acoustic and visual records, 39 were either detected or resighted in two or more aggregation seasons. Remarkably, five sharks initially tagged or photographed in 2010 were also detected in 2016. This interannual site fidelity is particularly interesting in the context of the 38 sharks tagged with both acoustic and satellite transmitters (S4 Table). Unfortunately, seven of these sharks never reported any satellite data. Another three were never tracked far from Shib Habil (maximum distance: 35–60 km) (Fig 5D), though this could be due to a combination of short deployment times (one shark was only tracked for 22 days) and the lack of archival data (the remaining two sharks were fitted with SPOT5 tags, so longer subsurface migrations may have gone undetected). Eleven sharks moved further from Shib Habil (maximum distance: 370–2826 km) (Fig 5D) but were never subsequently resighted within the aggregation, detected in the acoustic array, or satellite tracked near Shib Habil. This included three sharks that emigrated from the Red Sea entirely (Fig 5A). Finally, 17 sharks were tracked away from Shib Habil (maximum distance: 118–967 km) before eventually returning to the area (Fig 5B). Two of these sharks only returned to Shib Habil within the same year they were tracked away from it. The remaining 15 sharks recorded interannual homing-migrations, including four which returned to Shib Habil in multiple subsequent years (Fig 5C). Most tracked movements were confined to the southern central Red Sea, and activity was particularly concentrated around Shib Habil during the spring months associated with the aggregation (Fig 6).

## Discussion

Concurrent visual census, passive acoustic monitoring, and satellite telemetry of the same individual *R*. *typus* is unprecedented. The visual census and acoustic monitoring provide long term sightings dependent and independent assessments of the sharks’ residency patterns and spatial distribution. The satellite telemetry contributes information on dispersal behavior. Collectively, these data demonstrate a clear annual cycle of immigration, aggregation, and dispersal of *R*. *typus* at Shib Habil. This result would not be possible with any of the three methodologies individually: visual census and acoustic monitoring cannot conclusively demonstrate absence while satellite telemetry does not typically track an individual long enough to demonstrate interannual fidelity. This multimethod description of the Shib Habil aggregation provides a strong basis for comparison: comparing the present results to previous research from Shib Habil and comparing Shib Habil to other aggregations.

### Comparing results to previous work at Shib Habil

The passive acoustic results in this study largely corroborate, but also expand upon, previous sightings-based research at Shib Habil [28]. For instance, visual census records show high *R*. *typus* presence in March, April, and May, but the lack of survey effort at other times of the year make it impossible to judge the aggregation’s seasonality from sightings data alone. The continuous monitoring provided by the receiver array confirms high occupancy of *R*. *typus* from March to May, but also reveals moderate activity in January, February, and June as well as the relative absence of tagged sharks from July to December. Another example of the acoustic results agreeing with, but expanding on, the visual census is the similar mixed models derived from the two datasets. The strong seasonal influence and weak lag-effect shown for both methods suggest a high level of seasonal site fidelity. Despite similarities in model trends, the comparison also shows that the receiver array was far more reliable at detecting the presence of *R*. *typus*. While recapture probabilities projected by both models peak at roughly the same times, they are consistently and significantly higher for the acoustic monitoring (Fig 3). The difference in performance between the two methods reflects the ability of a well-maintained receiver array to monitor an area continuously and at depth. Visual census, on the other hand, is usually confined to daylight hours, surface waters, and limited survey effort.

Spatially, acoustic detections and visual encounters were both highly concentrated along the exposed side of Shib Habil and were modestly frequent on its sheltered side [28]. However, the receiver array also revealed another hotspot on the northern shelf. This additional site confirms the existence of high-use areas that are close to Shib Habil but outside the visual survey zone and suggests that there might be others beyond the range of the receiver array. This raises the possibility that annual declines in sightings and acoustic detections are caused by small-scale shifts to nearby, unmonitored habitat [14]. However, the satellite telemetry data shows most tracked sharks moving away from Shib Habil after the aggregation season and dispersing into the wider Red Sea. The motivations for these patterns of *R*. *typus* behavior are unknown [53]. Most sharks observed in visual surveys were engaged in active feeding [28], implying that patchy and ephemeral food resources may influence the seasonal presence of sharks at this site. However, other large planktivores tracked near Shib Habil do not exhibit any seasonal pattern in their use of the area, indicating that suitable food may be available year-round [54]. Another possibility is that the seasonal shift away from Shib Habil could be driven by changes in vertical behavior, with the sharks moving offshore to gain greater access to deep water. This has been shown for basking sharks (*Cetorhinus maximus)* in the western Atlantic [55], but is not supported by the archival depth data for *R*. *typus* at Shib Habil [13]. More research is clearly needed to identify the underlying causes of the aggregation, including those driving its seasonality and spatial distribution.

Finally, the acoustic detection record largely confirms the broad sexual parity and integration suggested by visual census [28]. The tagged population was evenly divided between males and females and there were no significant sexual differences in array-wide detection counts, days detected, or residence index values. The mixed-effects modeling did not find significant sexual influences on either acoustic or visual recapture probability. Sex was never found to have significant predictive value and the most likely models did not include sex as an explanatory variable. Sexual differences in spatial distribution were similarly modest. Three stations exhibited sexual differences which were significant at α = 0.05, but not at the Bonferroni corrected α = 0.0008. These three stations recorded relatively few detections, suggesting only limited use by either sex. The rest of the array, including all of the most frequently visited stations, reported statistically similar detection data and spatial index values for both male and female sharks. Overall, the acoustic record shows a high degree of spatiotemporal overlap and consistent shared habitat-use for male and female sharks at this site.

### Comparing Shib Habil to other aggregations

The general agreement between the acoustic and visual datasets at Shib Habil, especially with regard to the highly seasonal nature of the aggregation, is in stark contrast to the cryptic residency of *R*. *typus* reported at other sites [14, 39]. For example, the visual census record from Mafia Island initially appears very similar to the results at Shib Habil: many sightings during part of the year followed by months of apparent absence [14]. Passive acoustic results from Mafia, however, show many of the sharks remaining in the area year-round despite their disappearance from visual surveys. During the two year study in Mafia, at least 32% of tagged sharks were detected each month, producing a median R_min_ of 0.24 [14]. For comparison, the first two years of monitoring at Shib Habil included eight months in which fewer than 5% of tagged sharks were detected and produced a median R_min_ of 0.01. These patterns are also clear in the mixed-effects models from the two areas. Both GAMMs from Shib Habil, and the visual GAMM from Mafia show strong annual cycles and weak lag-effects on the odds of recapture. In contrast, acoustic recapture odds at Mafia were only weakly affected by time of year but declined monotonically with lag [14]. Both datasets (Mafia and Shib Habil) show seasonal changes in *R*. *typus* habitat selection: the two populations periodically move beyond the range of visual surveys. The discrepancy in the two sites’ acoustic records is caused by a difference of scale. Most sharks at Shib Habil move hundreds of kilometers away during the offseason, far beyond the range of the receiver array [13]. At Mafia, many of the sharks move a just few kilometers further from shore where they continue to be detected [14].

At Ningaloo Reef, the majority of all visual encounters occur in April, May, June, or July [39]. In contrast, acoustic activity is highest in September and October. Ningaloo’s acoustic record also shows a short offseason in February and March. This seasonal lull suggests that year-round residency at Ningaloo is less common than at Mafia Island. However, Ningaloo’s seasonal fluctuations are also not as pronounced as those from Shib Habil where nearly 50 percent of all detections are recorded in April while fewer than 2% are recorded in the six months from July through December. The intermediate results for Ningaloo are interesting, but somewhat preliminary due to poor tag retention [39]. Average monitoring periods (64.7 days), days detected (9.6), and R_max_ (0.18) at Ningaloo [39] are all less than the corresponding values from Shib Habil (304.05 days, 20 days, and 0.26 respectively). The continued monitoring and additional tagging at Ningaloo proposed by Norman et al. [39] could help resolve some of this ambiguity.

Despite their differences, the passive monitoring studies at Mafia, Ningaloo, and Shib Habil all support the importance of supplementing visual census with sightings-independent data [14, 39]. The seasonality and spatial distribution of most known aggregations have been described almost exclusively from encounter records. At many of these sites [25, 56] visual census records show clear annual patterns in sightings frequency, indicating residency behaviors similar to those shown for Shib Habil. However, similar studies have also suggested possible year-round residence in the Maldives [7], described aseasonal *R*. *typus* occurrence in Honduras [21], and shown the Galapagos to be a migratory waystation rather than an aggregation [12]. Research from Mozambique [11] and the Philippines [22] has also shown that habitat selection and residency patterns can shift in response to changes in the local environment or due to human influences. It is becoming increasingly clear that the movement ecology of *R*. *typus* may be site-specific, and identifying the characteristics of each aggregation could be vital to the conservation of these areas.

Another example of site specificity in *R*. *typus* ecology is the sexual parity shown here and in previous studies at this site [13, 28]. These demographics are unusual; only one other site (St. Helena, United Kingdom) has even preliminary evidence of attracting both sexes in roughly equal numbers [57] (www.whaleshark.org). Other *R*. *typus* aggregations are dominated either by immature males [7, 9, 15, 19, 22, 23, 30, 31, 58, 59, 60] or mature females [12, 60]. Three explanations have been proposed for the relative absence of immature females at most sites [60]. The first is that juvenile males and females have different preferred diets, leading to separate foraging grounds. Theevidence for this in the available data is limited. Male-dominated feeding aggregations are driven by a wide variety of plankton [4, 10, 61–64], suggesting that *R*. *typus* forage for areas of high prey density rather than targeting specific taxa [62]. Moreover, fatty acid analysis of *R*. *typus* tissue samples has not revealed significant sexual differences in diet [65]. Within Shib Habil, male and female *R*. *typus* forage in the same areas and are often observed feeding in close proximity, making it unlikely that they are targeting different food sources at this site [28]. Still, without identifying the exact prey being targeted at Shib Habil or gathering more information on the comparative diets of male and female *R*. *typus* from other locations, there are not enough data to eliminate this explanation. Sexual disparity might also be caused by males and females following different migratory routes [60]. While this may be true for mature *R*. *typus* [12, 66], there is little evidence to suggest that there are sex-related differences in the movements of juveniles. Satellite telemetry from Shib Habil revealed no sexual pattern in *R*. *typus* dispersal behavior and such a pattern would certainly be expected if the animals were on sexually-determined migrations [13]. The last potential explanation is that immature *R*. *typus* may be segregating based on sexual differences in temperature preference [60]. This possibility is intriguing given the evidence that thermoregulation is a strong driver of *R*. *typus* migration [67], vertical behavior [68], and physiology [69]. The Red Sea is thermally homogenous at depth with maximum surface temperatures of ~30°C and minimum temperatures at depth of ~22°C [70]. This 22°C isotherm extends from 200 m to more than 2000 m depth throughout the entire Red Sea [70]. If sexual segregation in *R*. *typus* is based on thermal habitat selection, then the consistently warm waters of the Red Sea may explain the integration found at Shib Habil.

## Conclusions

Both the photographic and acoustic histories show that Shib Habil attracts a seasonal aggregation of juvenile *R*. *typus* that tend to remain in the area for a few weeks or months before periods of prolonged absence. Incorporating the satellite data has demonstrated that many animals leave the area before returning in subsequent years. This combination of traits would seem to fulfill the criteria of a shark nursery [71]. However, due to the apparent absence of neonatal *R*. *typus* at this site, Shib Habil might be more accurately described as a staging ground for juveniles and sub-adults. Such areas are likely critical to the conservation of the species as a whole [72], which is especially relevant given the recent reclassification of *R*. *typus* as Endangered throughout its entire range [73]. While there does not appear to be a targeted *R*. *typus* fishery in the Red Sea [74], boat strikes have been identified as a potential threat to local populations [13, 28]. Recent bleaching events in the southern central Red Sea might also affect the sharks’ behavior [75]. The results of this study, along with previous work at Shib Habil [13, 28], have established an important historical baseline for directing additional research and by which to compare future fluctuations in the ecology of the aggregation.

With regard to other aggregations and even other species, this paper collects several tools for incorporating passive acoustic monitoring into photo-identification and satellite telemetry research. Visual census remains a vital component of *R*. *typus* study, but researchers should be aware of the method’s limitations and corroborate encounter records with other data where possible. High resolution, sightings-independent techniques like passive acoustic telemetry play an important role in establishing more accurate site descriptions and directing management efforts accordingly. Future passive acoustic studies targeting *R*. *typus* should focus on expanding the number of monitored aggregations as well as increasing the time series and tagged population-sizes for Shib Habil, Mafia Island, and Ningaloo Reef. Researchers should also work toward establishing standard analytical practices for acoustic detections of *R*. *typus*, especially for simple summary statistics like the residence indices. At Shib Habil the average difference between an individual’s R_min_ and its R_max_ was 0.21 (range: 0.00 to 1.00). The two metrics are clearly not interchangeable and calculating only one of them precludes easy comparison to studies using the other. One possible solution, as demonstrated here, is to calculate and report both. Finally, continued collaboration and data-sharing among scientists at different aggregations remains an essential aspect of *R*. *typus* research. Cooperative efforts have greatly increased the effectiveness of visual census and photo-identification studies [25] and similar approaches can also be applied to more expensive, telemetry-based data [76].

## Supporting information

S1 Appendix Detailed GAMM methodology. A step-by-step walkthrough of the generalized additive mixed effects modeling from uploading raw data to interpreting results.
(DOCX)

S1 Dataset Analyzed acoustic records. A spreadsheet containing all 35,571 acoustic detections used in this study.
(XLSX)

S2 Dataset Analyzed visual census records. A spreadsheet listing the photographic records of tagged sharks
(XLSX)

S1 Table Array metadata. Table listing all receiver stations used in this study along with summaries of each stations location, monitoring history, and detection record.
(PDF)

S2 Table Mixed-effects model selection. Table listing all candidate GAMMs and their AIC values. The models with the lowest AIC were selected and used to calculate the odds of acoustic/visual recapture.
(PDF)

S3 Table Selected GAMM results. Table listing the parameters of the selected GAMMs and summarizing their effect on the overall model.
(PDF)

S4 Table Tagged shark metadata. Table listing all tagged sharks and summarizing each individual’s acoustic, visual, satellite, and multimethod tracking data.
(PDF)
