# Supplementary material for: Multi-method assessment of whale shark (Rhincodon typus) residency, distribution, and dispersal behavior at an aggregation site in the Red Sea
Source: PLoS One. 2019 Sep 9;14(9):e0222285. doi: 10.1371/journal.pone.0222285 (PMC6733483; doi:10.1371/journal.pone.0222285)
Supplement: S1 Appendix — A step-by-step walkthrough of the generalized additive mixed effects modeling from uploading raw data to interpreting results. (DOCX) [file pone.0222285.s001.docx]

**S1 Appendix: detailed GAMM methodology**

**Introduction**

This procedure step-by-step guide to the Generalized Additive Mixed-effects Models (GAMMs) from the main text of this paper. It contains instructions for uploading and filtering data, model formulation, and interpretation of results. The whale shark passive acoustic and visual census data from the main text of this paper are used as a worked-through example, complete with the necessary R scripts. Readers wishing to follow along with the example analyses can find all of the necessary data and programming scripts [here](https://github.com/efcaguab/ws-allith-habitat-selection).

**Data**

**Acoustic monitoring**

The first step to any analysis is to acquire and format the necessary data. Building the desired GAMMs from passive acoustic detections requires several pieces of information including the raw detection record, the raw receiver events, the deployment history of the acoustic array, metadata for each tagged shark and receiver station, and information on known tag losses.

- The raw acoustic record is the CSV file exported from the Vemco User Environment (VUE) (see example below). For the Al Lith whale shark analysis this data is contained in the “AllDetections_Lith.csv” and the “2116 detections_Lith” files. In total, these files consist of 124322 detection records and contains ALL detections of tags which were eventually deployed on whale sharks in Al Lith, including detections from range tests and other extraneous records that need to be removed before the data can be analyzed.
- The raw events record is another VUE export containing the complete daily logs for every receiver. For the Al Lith whale shark analysis this file is listed as “AllEvents_Lith.csv” and consists of 406521 log entries. This file contains the complete daily logs for ALL receivers which were ever deployed in Al Lith, including logs from other studies at other sites. As with the raw detections, these data need to be filtered before being analyzed.
- The deployment history of the array is a record of when specific receivers were deployed to and retrieved from each station. For the Al Lith whale shark analysis this file is listed as “ArrayEvents_Lith.csv” and consists of 974 deployment/retrieval records.
- The shark metadata lists each detected tag and the characteristics of the shark on which it was deployed. The characteristics include animal sex, estimated size, tagging date, and tag number. For the Al Lith whale shark analysis this file is listed as “WSTags_Lith.csv” and includes the individual shark information for 100 tags that were detected in the array.
- The station metadata lists all deployed stations and their characteristics, including geographic coordinates and the depth of the attached receiver. For the Al Lith whale shark analysis this file is listed as “Stations_Lith.csv”.
- Known tag losses lists all sharks known to have lost their tags over the course of the study, when they were first resighted without their tag, their date of last detection, and whether those sharks were eventually retagged. For the Al Lith whale shark analysis this file is listed as “shed_tag_lith2.csv” and includes information on 11 sharks which shed their original tags over the course of the study.

**Visual census**

Building the GAMMs based on visual census required the raw encounter data as well as a record of survey effort.

- The raw encounter data is a CSV file downloaded from the Wildbook for Whale Sharks online database (www.whaleshark.org) which lists all reported whale shark encounters for a specific area. For the Al Lith whale shark analysis this file is listed as “visual_sightings.csv” and consists of 356 encounter records from around Shib Habil. The file includes ALL encounter records, even those of untagged sharks, and needs to be filtered before analysis.
- The survey effort record is a list of days and times spent searching for the target species. For the Al Lith whale shark analysis this file is listed as “Survey_Effort_Lith.csv” and includes information on 268 shark surveys at Shib Habil.

**Uploading and filtering data**

After the necessary data has been acquired and formatted. The next step is to upload the files onto R and to refine the raw data for analysis. This includes removing extraneous detections from the acoustic record, removing extraneous encounters from the visual census data, correcting receiver records for temporal drift/ time-zone inconsistencies, deriving effort from receiver deployment/survey records, and accounting for known tag losses.

The code for uploading and the acoustic data can be found in “01_prepare_data.R” while the code for the visual data is available under “11_prepare_data_visual.R”. These same scripts also perform most of the data filtering including removing extraneous sightings/detections, correcting receiver times, and accounting for effort. Known tag losses are taken into account in the “02_calculate_lags.R” script.

**Model formulation**

**Calculating explanatory variables**

**Smoothed variables**

Smoothed variables included temporal lag and time-of-year. Lag was quantified as the set of all possible time-differences between potential capture events for a given animal. Time-of-year was quantified as week of the year, so sightings/detections from January 1^st^ through the 7^th^ would have a time-of-year value equal to 1, January 8^th^-15^th^ would have a value of 2 and so on. Both of these terms were included as smooths in the models. Time of the year was included as a cyclic cubic regression spline (so that the value at the end of the year to matches the value at the beginning of the year). Lag was included as a low rank isotropic smoother, which is the default in our modelling framework.

**Linear variables**

Size was based off of the visual estimates reported for each shark. All estimates were rounded to the nearest half meter. Sex was quantified as 1 if claspers were present (male) and 0 if they were absent (female). Survey Effort was quantified differently for the visual and acoustic GAMMs. Visual census effort was quantified as the number of survey hours per week and acoustic effort was quantified as the weekly average number of receivers in the inshore and offshore arrays.

**Random variables and error**

Using every possible pair of potential capture events allows us to compute a lag effect which is not influenced by time-of-year, but also introduces pseudo-replication into the model. Fortunately, the resulting pseudo-correlation can easily be accounted for by using the date of first capture (initial photographic/acoustic tagging) as a random variable. Similarly, the effects of individual variation can be accounted for by including shark identification numbers as another random variable. The final explanatory variable was error, which is given a binomial structure to match the response variable.

**Calculating the response variable**

The filtered data can now be properly analyzed. Our approach quantifies animal residency as a binomial occupancy metric for each individual. These calculations are performed independently for each shark’s visual census and passive acoustic datasets and are based on the following pair of equations:

$$\vec{F}\left( S_{i},\tau\right)=[\mathbb{G}\left( S_{i},j+\tau\right)]_{j=1}^{N-\tau} \text{where} S_{i}\in\sigma_{j}$$

$$\mathbb{G}\left( S_{i},k \right)=\left\{ \begin{aligned} 1 \text{if} S_{i}\in\sigma_{k} \\ 0 \text{otherwise} \end{aligned} \right.$$

Where is 𝑁 the number of surveys performed since the individual $S_{i}$ was first captured and the corresponding presence-absence vector 𝐹 is defined for each set of lags 𝜏, where 𝜏 is the difference between the previous recapture event 𝑗 and the posterior recapture event 𝑘, as 1 if the individual belongs to the set of individuals recaptured on both occasions ($\sigma_{j}$ and $\sigma_{k}$) and defined as 0 otherwise. Due to the binomial nature of the occupancy metric, we use the canonical logit linking function to define the relationship between the response and explanatory variables.

Acoustic occupancy and temporal lag were calculated via the “pres.abs.lag.R” function embedded in the “02_calculate_lags.R” script. The code for calculating the other explanatory variables for the acoustic GAMM is included in the “01_prepare_data.R” script. All variables for the visual GAMM, including lag and occupancy, are calculated within the “11_prepare_data_visual.R” script.

**Fitting the models**

Now that we have the general form of the models, the permutations can be fit to the visual and acoustic data. Every possible permutation that includes both smoothed terms (temporal lag and time-of-year) is assessed in this way. The random and fixed effects were fit separately for each GAMM. The scripts for fitting the acoustic GAMMs are listed as “03 acoustic_models_ random_effects.R” and “ 04_acoustic_models_fixed_effects.R”. The scripts for the visual GAMMs are listed as “ 12_visual_models_random_effects.R” and “ 13_visual_models_fixed_effects.R”.

**Model selection and interpretation of results**

**Selection by AIC**

Once all model permutations are fit, the most parsimonious visual and acoustic GAMMs can be selected based on AIC. The selected visual GAMM included lag, time-of-year, size, and survey effort as explanatory variables. The selected acoustic GAMM included lag, time-of-year, size, and survey effort as explanatory variables (S2 Table).

**Calculating recapture odds/probabilities**

The values predicted by the selected GAMMs are the odds of acoustic/visual recapture. Odds show how the likelihood of recapture fluctuates relative to a baseline, allowing us to estimate the effect of specific explanatory variables in isolation. For instance, in Figure 2A we can show the effect of time-of-year on the likelihood of recapture without the confounding influence of temporal lag. In Figure 2B we reverse this and show the effects of lag without considering time-of-year.

In order to better understand the combined effects of the different explanatory variables, we convert the odds into probabilities by summing the predicted values for all variables along with the intercept and applying an inverse logit function. For example, in Figure 3 we are able to show that the combined effects of temporal lag and time-of-year produce an annual cycle of high and low recapture probabilities. The code for model selection and interpretation can be found within the “outputs.R” script.
