# Supplementary material for: Multi-method assessment of whale shark (Rhincodon typus) residency, distribution, and dispersal behavior at an aggregation site in the Red Sea
Source: PLoS One. 2019 Sep 9;14(9):e0222285. doi: 10.1371/journal.pone.0222285 (PMC6733483; doi:10.1371/journal.pone.0222285)
Supplement: S1 Table — Table listing all receiver stations used in this study along with summaries of each stations location, monitoring history, and detection record. (PDF) [file pone.0222285.s004.pdf]

Station Metadata				Raw Detection Data				Spatial Residence (Rspatial)	
Name	Region	Latitude	Longitude	Total Days Monitored	Total Detections	Detections per Male	Detections per Female	Male Mean	Female Mean
E1	Exposed Shib Habil	20.12920	40.20968	1911	11275	100.667	203.629	0.509	0.412
E2	Exposed Shib Habil	20.11847	40.21453	1744	3278	55.444	64.933	0.333	0.320
E3	Exposed Shib Habil	20.11115	40.21888	1269	1387	47.923	46.150	0.312	0.335
E4	Exposed Shib Habil	20.10055	40.22643	1897	2910	20.800	55.143	0.215	0.183
E5	Exposed Shib Habil	20.08855	40.23510	1218	1964	25.429	41.909	0.258	0.255
Sh1	Exposed Shib Habil	20.12632	40.21617	1530	3422	15.125	48.567	0.247	0.231
Sh2	Sheltered Shib Habil	20.12353	40.22435	2233	1363	22.944	13.971	0.105	0.068
Sh3	Sheltered Shib Habil	20.12155	40.21977	1631	1955	43.040	28.667	0.101	0.076
Sh4	Sheltered Shib Habil	20.11642	40.22397	884	1363	29.909	26.692	0.092	0.079
Sh5	Sheltered Shib Habil	20.10033	40.23085	1159	170	2.333	4.214	0.035	0.064
Sh6	Sheltered Shib Habil	20.10913	40.22597	1644	473	5.719	8.029	0.052	0.053
I1	Inshore	20.11913	40.23922	1604	172	1.813	3.030	0.025	0.025
I2	Inshore	20.10877	40.25383	1007	29	0.565	0.571	0.002	0.008
I3	Inshore	20.09453	40.27482	1050	0	0	0	0	0
N1	North Shelf	20.13787	40.22172	1647	188	3.538	3.100	0.020	0.009
N2	North Shelf	20.17630	40.09757	794	135	3.524	1.577	0.046*	0.01*
N3	North Shelf	20.13343	40.10148	1106	31	0.905	0.357	0.012	0.005
N4	North Shelf	20.16568	40.16773	1650	4813	75.607	74.212	0.287	0.251
N5	North Shelf	20.17305	40.08152	1238	1060	32.000	12.321	0.143	0.078
S1	South Shelf	20.05742	40.37817	2152	0	0	0	0	0
S2	South Shelf	20.03470	40.35413	884	55	1.773	0.615	0.003	0.020
S3	South Shelf	20.03507	40.39563	1109	8	0.348	0.000	0.001	0.000
S4	South Shelf	20.00517	40.39945	603	17	0.857	0.364	0.002	0.012
S5	South Shelf	20.01630	40.41815	883	20	3.524	0.077	0.012	0.001
S6	South Shelf	19.98447	40.40625	638	781	5.059	22.409	0.030	0.086
S7	South Shelf	19.99183	40.41287	643	129	0.647*	2.818*	0.001*	0.047*
A1	Abu Latt	19.94527	40.15162	794	29	0.765	0.609	0.008	0.011
A2	Abu Latt	19.96717	40.15167	690	34	0.105*	1.391*	0.027	0.048
A3	Abu Latt	19.98308	40.13472	878	82	3.095	0.654	0.044	0.022
O1	Offshore	19.78722	39.95393	582	2	0	0.143	0	0.012
O10	Offshore	19.78738	39.95207	581	2	0.083	0.214	0.014	0.012
O11	Offshore	19.76375	39.95963	377	0	0	0.333	0	0.002
O12	Offshore	19.76247	39.96040	357	0	0	0.167	0	0.002
O13	Offshore	19.77957	39.94128	373	1	0	0.286	0	0.017
O14	Offshore	19.78392	39.94237	372	0	0	0	0	0
O15	Offshore	19.78180	39.94210	594	0	0	0	0	0
O16	Offshore	19.76888	39.96967	595	0	0	0	0	0
O17	Offshore	19.77008	39.97263	582	0	0	0	0	0
O18	Offshore	19.76778	39.97068	583	3	0	0.143	0	0.012
O19	Offshore	19.76408	39.89020	379	2	0	0	0	0
O2	Offshore	19.78758	39.95578	582	4	0	0	0	0
O20	Offshore	19.76745	39.89238	581	9	0.125	0	0.006	0
O21	Offshore	19.77408	39.89265	581	16	0	0	0	0
O22	Offshore	19.78055	39.89298	260	3	0	0	0	0
O23	Offshore	19.75210	39.90915	620	16	0	0	0	0
O24	Offshore	19.75100	39.90748	387	14	0	0	0	0
O25	Offshore	19.75020	39.90563	610	26	0	0.214	0	0.001
O26	Offshore	19.74928	39.90415	610	16	0.250	0	0.031	0
O27	Offshore	19.74778	39.90418	372	4	0	0.643	0	0.013
O28	Offshore	19.74728	39.90620	611	34	0	0.857	0	0.073
O29	Offshore	19.74747	39.90810	382	30	0	0.273	0	0.001
O3	Offshore	19.78898	39.95667	393	4	0.333	0.857	0.021	0.073
O30	Offshore	19.74818	39.90973	621	34	0.818	0.417	0.033	0.002
O31	Offshore	19.74968	39.91112	621	14	1.250	0.714	0.042	0.074
O32	Offshore	19.75102	39.91173	621	36	0.750	0.500	0.007	0.072
O33	Offshore	19.75238	39.91145	620	19	0	0.400	0	0.035
O34	Offshore	19.75255	39.90998	583	23	0.750	1.500	0.021	0.110
O4	Offshore	19.78983	39.95533	393	2	0.818	1.750	0.048	0.133
O5	Offshore	19.79035	39.95350	619	4	0.750	1.500	0.035	0.030
O6	Offshore	19.79047	39.95217	619	0	0.417	0.429	0.021	0.015
O7	Offshore	19.79035	39.95042	523	0	0.833	1.500	0.021	0.088
O8	Offshore	19.78960	39.94962	619	0	0.583	0.786	0.021	0.088
O9	Offshore	19.78805	39.95032	392	0	0.500	1.214	0.035	0.073
