# Supplementary material for: Multi-method assessment of whale shark (Rhincodon typus) residency, distribution, and dispersal behavior at an aggregation site in the Red Sea
Source: PLoS One. 2019 Sep 9;14(9):e0222285. doi: 10.1371/journal.pone.0222285 (PMC6733483; doi:10.1371/journal.pone.0222285)
Supplement: S2 Table — Table listing all candidate GAMMs and their AIC values. The models with the lowest AIC were selected and used to calculate the odds of acoustic/visual recapture. (PDF) [file pone.0222285.s005.pdf]

Acoustic GAMM Selection			
**Model**	**Log-Likelihood**	**AIC**	**ΔAIC**
season + lag + effort (IS) + effort (OS) + size	-1763.9	3543.9	0
season + lag + effort (IS) + effort (OS) + sex + size	-1763.1	3546.2	2.37
season + lag + effort (IS) + size	-1767.2	3548.4	4.54
season + lag + effort (IS) + sex + size	-1766.4	3550.8	6.94
season + lag + effort (OS) + size	-1882.8	3779.6	235.74
season + lag + effort (OS) + sex + size	-1881.8	3781.7	237.79
season + lag + effort (IS) + effort (OS)	-1952.2	3918.5	374.63
season + lag + effort (IS)	-1953.9	3919.9	376.02
season + lag + effort (IS) + effort (OS) + sex	-1951.1	3920.2	376.36
season + lag + effort (IS) + sex	-1952.4	3920.7	376.87
season + lag + size	-1981.8	3975.7	431.81
season + lag + sex + size	-1980.6	3977.2	433.37
season + lag + effort (OS)	-2100.4	4212.9	668.99
season + lag + effort (OS) + sex	-2098.7	4213.3	669.43
season + lag	-2207.1	4424.2	880.29
season + lag + sex	-2205.2	4424.4	880.49

Visual GAMM Selection			
**Model**	**Log-Likelihood**	**AIC**	**ΔAIC**
season + lag + size	-22017.5	44049	0
season + lag + effort + size	-22112.4	44240.8	191.72
season + lag	-25600	51212.1	7163.08
season + lag + effort	-25685	51383.9	7334.91
season + lag + sex	-2.84E+17	5.67E+17	5.67E+17
season + lag + effort + sex	-2.84E+17	5.67E+17	5.67E+17
season + lag + sex + size	NA	NA	NA
season + lag + effort + sex + size	NA	NA	NA
