# Supplementary material for: Multi-method assessment of whale shark (Rhincodon typus) residency, distribution, and dispersal behavior at an aggregation site in the Red Sea
Source: PLoS One. 2019 Sep 9;14(9):e0222285. doi: 10.1371/journal.pone.0222285 (PMC6733483; doi:10.1371/journal.pone.0222285)
Supplement: S3 Table — Table listing the parameters of the selected GAMMs and summarizing their effect on the overall model. (PDF) [file pone.0222285.s006.pdf]

Selected Acoustic GAMM Results					
**Parameter**	**Estimated Degrees of Freedom**	**Estimated Value**	**Standard Error**	**Test Statistic**	**p-value**
Intercept	NA	-11.74	1.85	-6.35	0.00
Effort (IS)	NA	0.17	0.01	14.09	0.00
Effort (OS)	NA	0.01	0.00	2.95	0.01
Size	NA	0.53	1.15	1.46	0.25
Time of year	329.07	NA	NA	6.66	0.00
Temporal lag	88.70	NA	NA	7.39	0.00
**Selected Visual GAMM Results**					
**Parameter**	**Estimated Degrees of Freedom**	**Estimated Value**	**Standard Error**	**Test Statistic**	**p-value**
Intercept	NA	-5.68	-4.73	-4.73	0.00
Size	NA	0.19	0.66	0.66	0.51
Time of year	9.65	NA	NA	3.65	0.00
Temporal lag	12.95	NA	NA	1.00	0.00
