# Supplementary material for: Multi-method assessment of whale shark (Rhincodon typus) residency, distribution, and dispersal behavior at an aggregation site in the Red Sea
Source: PLoS One. 2019 Sep 9;14(9):e0222285. doi: 10.1371/journal.pone.0222285 (PMC6733483; doi:10.1371/journal.pone.0222285)
Supplement: S4 Table — Table listing all tagged sharks and summarizing each individual’s acoustic, visual, satellite, and multimethod tracking data. (PDF) [file pone.0222285.s007.pdf]

Shark Metadata			Passive Acoustic Montioring							Visual Census		Satellite Telemetry			Multimethod Tracking			
ID	Sex	Size (m)	Acoustic Tag Date(s)	Days Detected	Total Detections	Min Monitoring Period (days)	Max Monitoring Period (days)	Rmin	Rmax	Wildbook ID	Wildbook Encounters	Satellite Tag Date	Track Duration (days)	Cumulative Track Distance (km)	Combined Track Duration (days)	Combined Track Distance (km)	Max Distance to Aggregation	Migration Behavior
WS 001	NA	NA	3/27/2010	2	47	7	2233	0.001	0.286	R-161	1	3/27/2010	103	2826	103	2868	2826	Emigration
WS 002	NA	NA	3/28/2010	22	573	385	2233	0.010	0.057	R-162	1	3/28/2010	16	117	386	304	35	Short Track
WS 003	NA	4.5	3/28/2010	24	190	432	2233	0.011	0.056	R-165	1	3/28/2010	148	584	433	831	277	Interseasonal Return
WS 005	NA	4	3/29/2010	6	63	1042	2233	0.003	0.006	R-163	1	3/29/2010	156	56	1042	141	60	Short Track
WS 008	Female	3	4/30/2010	12	369	14	2201	0.005	0.857	R-035	4	3/29/2010	DNR	DNR	NA	NA	NA	NA
WS 009	Female	6.5	3/30/2010	4	60	2216	2232	0.002	0.002	R-031	2	3/29/2010	DNR	DNR	NA	NA	NA	NA
WS 011	NA	NA	3/30/2010	17	826	26	2232	0.008	0.654	R-166	1	3/30/2010	102	1493	107	1493	923	Emigration
WS 014	Male	3.5	4/12/2010	113	2024	683	2219	0.051	0.165	R-036	4	4/12/2010	265	2760	683	3677	547	Interseasonal Return
WS 015	Female	3.5	4/23/2010<br>3/16/2012	119	3301	528	1947	0.061	0.225	R-089	5	4/12/2010	197	2475	798	3358	538	Interseasonal Return
WS 016	Male	5	4/12/2010	15	114	1463	2219	0.007	0.010	R-167	1	4/12/2010	263	2699	1463	3174	633	Interseasonal Return
WS 018	Male	4	4/15/2010	0	0	NA	NA	NA	NA	R-041	1	4/12/2010	37	330	37	684	370	Emigration
WS 019	Female	3	4/15/2010	3	28	33	2216	0.001	0.091	R-094	2	4/12/2010	177	3448	180	3448	1181	Emigration
WS 020	Female	4	4/12/2010	7	134	360	2219	0.003	0.019	R-084	2	3/31/2011	68	1092	419	1180	287	Interseasonal Return
WS 023	Male	NA	4/15/2010	1	57	2	2216	0	0.500	R-169	1	4/15/2010	DNR	DNR	NA	NA	NA	NA
WS 024	Female	3	5/4/2010	7	859	294	2197	0.003	0.024	R-042	4	5/4/2010	162	2638	303	2856	967	Interseasonal Return
WS 025	Female	4	4/28/2010<br>4/20/2011	44	1325	412	2203	0.020	0.107	R-092	6	4/15/2010	DNR	DNR	NA	NA	NA	NA
WS 026	NA	NA	4/16/2010	6	46	657	2215	0.003	0.182	R-171	1	4/16/2010	259	3475	657	3841	320	Interseasonal Return
WS 027	NA	3	4/16/2010	2	30	11	2215	0.001	0.009	R-168	1	4/16/2010	167	3177	169	3177	1402	Emigration
WS 029	Female	7	4/16/2010	0	0	NA	NA	NA	NA	R-172	1	4/16/2010	DNR	DNR	NA	NA	NA	NA
WS 030	Male	3	5/4/2010	22	278	699	2197	0.010	0.031	R-177	1	5/4/2010	193	3000	700	3000	303	Interseasonal Return
WS 031	Male	3.5	5/4/2010	53	1350	365	365	0.145	0.145	R-037	4	5/4/2010	157	2438	366	2438	392	Interseasonal Return
WS 032	Male	NA	4/15/2010	62	751	1802	2216	0.028	0.034	R-040	3	NA	NA	NA	NA	NA	NA	NA
WS 033	Female	4.5	4/16/2010	265	3781	2213	2215	0.120	0.120	R-058	11	NA	NA	NA	NA	NA	NA	NA
WS 034	Male	3	4/22/2010	1	9	2	2209	0	0.500	R-034	1	4/22/2010	118	647	120	647	532	Emigration
WS 035	Male	4	4/22/2010<br>3/31/2016	9	378	290	322	0.028	0.031	R-124	4	4/22/2010	100	483	2177	779	381	Interseasonal Return
WS 036	Male	3.5	4/22/2010	1	17	3	2209	0	0.333	R-174	1	4/22/2010	17	53	22	56	35	Short Track
WS 037	Male	7	4/22/2010	2	14	4	2209	0.001	0.500	R-173	1	4/22/2010	82	760	88	760	716	Emigration
WS 038	Female	3	4/22/2010	8	343	282	2209	0.004	0.028	R-038	2	NA	NA	NA	NA	NA	NA	NA
WS 039	Female	4.5	4/27/2010	0	0	NA	NA	NA	NA	R-100	1	NA	NA	NA	NA	NA	NA	NA
WS 040	Male	4	4/28/2010	0	0	NA	NA	NA	NA	R-175	1	NA	NA	NA	NA	NA	NA	NA
WS 041	Female	4	4/30/2010	2	123	25	2201	0.001	0.080	R-176	1	NA	NA	NA	NA	NA	NA	NA
WS 042	Male	3	5/4/2010	36	666	61	2197	0.016	0.590	R-090	3	NA	NA	NA	NA	NA	NA	NA
WS 043	Female	3.5	5/7/2010	2	4	13	2194	0.001	0.154	R-096	1	NA	NA	NA	NA	NA	NA	NA
WS 044	Female	4	5/11/2010	3	22	3	2190	0.001	1.000	R-039	1	NA	NA	NA	NA	NA	NA	NA
WS 045	Male	3.5	5/11/2010	9	148	300	2190	0.004	0.030	R-099	3	NA	NA	NA	NA	NA	NA	NA
WS 046	Male	3	5/12/2010	6	26	7	2189	0.003	0.857	R-086	3	NA	NA	NA	NA	NA	NA	NA
WS 047	NA	4.5	5/14/2010	0	0	NA	NA	NA	NA	R-178	1	NA	NA	NA	NA	NA	NA	NA
WS 103	Male	4	3/31/2011<br>4/18/2012	115	3995	602	1844	0.062	0.191	R-070	7	3/31/2011	184	1142	951	1931	447	Interseasonal Return
WS 104	Female	3.5	4/2/2011<br>4/20/2012	46	812	101	1653	0.028	0.455	R-085	4	3/31/2011	175	1897	721	2487	592	Interseasonal Return
WS 105	Male	5	4/2/2011	0	0	NA	NA	NA	NA	R-180	1	4/2/2011	182	180	182	1535	402	Emigration
WS 106	NA	NA	4/2/2011	0	0	NA	NA	NA	NA	R-078	1	3/31/2011	DNR	DNR	NA	NA	NA	NA
WS 107	Female	4.5	4/2/2011<br>3/31/2016	50	1615	67	3883	0.013	0.746	R-073	11	4/2/2011	DNR	DNR	NA	NA	NA	NA
WS 108	Female	4.5	4/6/2011	2	15	3	1860	0.001	0.667	R-181	2	4/4/2011	281	3828	281	3828	592	Emigration
WS 109	Female	4	4/7/2011	23	473	361	1859	0.012	0.064	R-182	2	4/7/2011	192	3666	361	4102	598	Interseasonal Return
WS 110	Female	4.5	4/7/2011	15	367	355	1859	0.008	0.042	R-183	2	NA	NA	NA	NA	NA	NA	NA
WS 111	Male	4	4/16/2011	2	156	7	1850	0.001	0.286	R-102	2	NA	NA	NA	NA	NA	NA	NA
WS 112	Male	3.5	4/14/2011<br>4/18/2011	3	51	4	1848	0.002	0.750	R-098	5	NA	NA	NA	NA	NA	NA	NA
WS 113	Male	NA	4/14/2011	3	14	356	1852	0.002	0.008	R-185	1	NA	NA	NA	NA	NA	NA	NA
WS 114	Female	3.5	4/14/2011	0	0	NA	NA	NA	NA	R-186	1	NA	NA	NA	NA	NA	NA	NA
WS 115	Female	4	4/14/2011	27	213	588	1851	0.015	0.046	NA	NA	NA	NA	NA	NA	NA	NA	NA
WS 116	Female	4	4/14/2011	2	9	456	1852	0.001	0.004	NA	NA	NA	NA	NA	NA	NA	NA	NA
WS 117	Male	3.5	4/15/2011	4	166	5	1851	0.002	0.800	R-184	3	NA	NA	NA	NA	NA	NA	NA
WS 118	NA	3	4/20/2011	4	42	4	1846	0.002	1.000	NA	NA	4/17/2011	61	708	64	708	356	Emigration
WS 119	Male	4	4/18/2011	0	0	NA	NA	NA	NA	NA	NA	4/18/2011	70	65	70	1046	315	Intra-Seasonal Return
WS 120	Female	3.5	4/19/2011	48	961	450	1847	0.026	0.107	NA	NA	4/19/2011	78	635	450	970	118	Interseasonal Return
WS 121	Female	3.5	4/19/2011	3	16	5	1847	0.002	0.600	NA	NA	4/19/2011	165	1107	166	1107	391	Emigration
WS 123	Male	3.5	4/19/2011	9	562	349	1847	0.005	0.026	NA	NA	4/19/2011	99	1384	349	1813	503	Interseasonal Return
WS 124	Male	4.5	4/20/2011	0	0	NA	NA	NA	NA	R-197	1	4/20/2011	25	83	25	307	123	Intra-Seasonal Return
WS 126	Male	3	4/22/2011	5	109	24	1844	0.003	0.208	R-190	2	NA	NA	NA	NA	NA	NA	NA
WS 127	Female	6	4/22/2011<br>3/25/2014	3	159	4	780	0.004	0.750	R-115	2	NA	NA	NA	NA	NA	NA	NA
WS 128	NA	3.5	4/22/2011	16	90	69	1844	0.009	0.232	NA	NA	NA	NA	NA	NA	NA	NA	NA
WS 129	Male	4.5	4/26/2011	26	326	683	1840	0.014	0.038	NA	NA	NA	NA	NA	NA	NA	NA	NA
WS 130	Female	3.5	4/26/2011	2	18	351	1840	0.001	0.006	R-193	1	NA	NA	NA	NA	NA	NA	NA
WS 131	Female	3	4/27/2011	4	48	326	1839	0.002	0.012	R-194	1	NA	NA	NA	NA	NA	NA	NA
WS 202	Male	5	3/16/2012	13	65	664	1515	0.009	0.020	R-196	1	NA	NA	NA	NA	NA	NA	NA
WS 203	Male	3	3/16/2012	24	227	367	1515	0.016	0.065	R-199	2	NA	NA	NA	NA	NA	NA	NA
WS 204	Male	3	3/29/2012	8	233	366	1502	0.005	0.022	R-132	2	NA	NA	NA	NA	NA	NA	NA
WS 205	NA	3	4/19/2012	5	17	46	1481	0.003	0.109	R-135	1	NA	NA	NA	NA	NA	NA	NA
WS 206	Male	3.5	4/19/2012	28	448	367	1481	0.019	0.076	R-134	2	NA	NA	NA	NA	NA	NA	NA
WS 207	Female	3	4/19/2012	11	59	366	1481	0.007	0.030	R-201	1	NA	NA	NA	NA	NA	NA	NA
WS 208	Female	4	4/19/2012	15	123	37	1481	0.010	0.405	R-200	1	NA	NA	NA	NA	NA	NA	NA
WS 209	Female	4	5/3/2012	26	553	346	1467	0.018	0.075	R-101	2	NA	NA	NA	NA	NA	NA	NA
WS 210	NA	NA	5/3/2012	4	30	270	1467	0.003	0.015	R-203	1	NA	NA	NA	NA	NA	NA	NA
WS 211	Male	4.5	5/3/2012	3	35	678	1467	0.002	0.004	R-202	1	NA	NA	NA	NA	NA	NA	NA
WS 212	Female	4	5/4/2012	6	57	320	1466	0.004	0.019	R-189	1	NA	NA	NA	NA	NA	NA	NA
WS 401	NA	6.5	4/11/2014	4	33	10	759	0.005	0.400	R-116	1	NA	NA	NA	NA	NA	NA	NA
WS 402	Female	4.5	4/11/2014<br>3/10/2016	42	786	463	465	0.090	0.091	R-061	6	NA	NA	NA	NA	NA	NA	NA
WS 403	Male	4	4/12/2014	19	269	423	900	0.021	0.045	DJ-109	2	NA	NA	NA	NA	NA	NA	NA
WS 404	Female	5	4/26/2014	2	59	6	744	0.003	0.333	R-121	1	NA	NA	NA	NA	NA	NA	NA
WS 501	Female	5	4/19/2015	2	7	15	386	0.005	0.133	R-064	3	NA	NA	NA	NA	NA	NA	NA
WS 502	Male	3.5	4/19/2015	3	22	22	386	0.008	0.136	R-136	2	NA	NA	NA	NA	NA	NA	NA
WS 503	Male	5	4/28/2015	2	12	64	377	0.005	0.031	R-142	1	NA	NA	NA	NA	NA	NA	NA
WS 504	Male	NA	4/28/2015	0	0	NA	NA	NA	NA	R-141	1	NA	NA	NA	NA	NA	NA	NA
WS 505	Female	4	4/28/2015	59	1739	374	377	0.156	0.158	R-072	10	NA	NA	NA	NA	NA	NA	NA
WS 506	Female	6	4/28/2015	9	155	20	377	0.024	0.450	R-138	1	NA	NA	NA	NA	NA	NA	NA
WS 507	Male	6	4/28/2015	15	498	353	377	0.040	0.042	R-103	7	NA	NA	NA	NA	NA	NA	NA
WS 508	Male	3.5	4/30/2015	3	160	12	375	0.008	0.250	R-059	2	NA	NA	NA	NA	NA	NA	NA
WS 509	Male	NA	5/2/2015	14	125	370	373	0.038	0.038	R-105	2	NA	NA	NA	NA	NA	NA	NA
WS 510	Male	3.5	5/9/2015	1	5	13	366	0.003	0.077	R-106	2	NA	NA	NA	NA	NA	NA	NA
WS 601	Male	4	3/19/2016	0	0	NA	NA	NA	NA	R-082	1	NA	NA	NA	NA	NA	NA	NA
WS 603	Male	4.5	3/31/2016	28	822	37	39	0.718	0.757	R-074	9	NA	NA	NA	NA	NA	NA	NA
WS 604	Female	7	3/31/2016	0	0	NA	NA	NA	NA	R-107	1	NA	NA	NA	NA	NA	NA	NA
WS 605	Female	4.5	4/3/2016	27	1653	35	36	0.750	0.771	R-075	11	NA	NA	NA	NA	NA	NA	NA
WS 606	Male	3.5	4/3/2016	3	25	3	36	0.083	1.000	R-123	1	NA	NA	NA	NA	NA	NA	NA
WS 607	Male	NA	4/9/2016	7	46	26	30	0.233	0.269	R-126	4	NA	NA	NA	NA	NA	NA	NA
WS 608	Female	3	4/24/2016	10	862	11	15	0.667	0.909	R-081	3	NA	NA	NA	NA	NA	NA	NA
WS 609	Male	3.5	5/1/2016	7	163	8	8	0.875	0.875	R-108	4	NA	NA	NA	NA	NA	NA	NA
